# Practical Points

**Published:** 1897-10-16

**Authors:** 


### PRACTICAL POINTS.

*Sunday Funds and Nursing Associations.—"A Correspondent" writes: Could you tell me of any district nursing associations which receive a grant from the Hospital Saturday or Sunday Funds? The committee of one local association are applying for such a grant, and are told that the application would be more favourably received if precedents for such a grant could be quoted. I should be glad also to know the amounts granted.*

The contributions raised from Hospital Sunday in London are distributed amongst hospitals and dispensaries only which are managed by committees duly constituted and fulfilling the regulations laid down by the Council. The Hospital Saturday Fund in London makes grants to institutions for the gratuitous nursing of the sick poor in their own homes. Twelve such institutions received grants in 1895, varying in amount from £130 to the East London Nursing Association to £12 granted to that at Woolwich. The practice of making grants to district nursing associations by central funds varies, therefore, as you may conclude from these instances, according to the views held by those who are responsible for their distribution. A great deal may be said on both sides of this question, and the decision must in each case be governed by local circumstances, the quality of the work done, and the amount of funds at the disposal of the central fund.

*The Cost of a Hospital.—"A Correspondent" writes: I shall feel grateful if you will be good enough to give me some information respecting the cost of building a hospital for seamen. It is proposed to build a hospital to contain accommodation for sixty patients, all of whom would, of course, be males. Provision would have to be made for the residence of the resident medical officer and his family. There would have to be a room for the out-patients.*

The cost of such a hospital as you propose in London would be about £300 per bed, but in the country it should be possible to secure a thoroughly efficient hospital at an expenditure of £200 per bed. The elimination of one sex, of course, should make the planning less expensive and elaborate. In "Burdett's Cottage Hospitals" (third edition) there are some model plans given, with particulars, and, if it is not proposed to build a large hospital, some of these plans may be found of use.

